# Lack of the Lysosomal Membrane Protein, GLMP, in Mice Results in Metabolic Dysregulation in Liver

**DOI:** 10.1371/journal.pone.0129402

**Published:** 2015-06-05

**Authors:** Xiang Yi Kong, Eili Tranheim Kase, Anette Herskedal, Camilla Schjalm, Markus Damme, Cecilie Kasi Nesset, G. Hege Thoresen, Arild C. Rustan, Winnie Eskild

**Affiliations:** 1 Department of Bioscience, University of Oslo, Oslo, Norway; 2 Department of Pharmaceutical Biosciences, School of Pharmacy, University of Oslo, Oslo, Norway; 3 Institute of Biochemistry, Christian-Albrechts-Universität Kiel, Kiel, Germany; 4 Department of Pharmacology, Institute of Clinical Medicine, Faculty of Medicine, University of Oslo and Oslo University Hospital, Oslo, Norway; The Chinese University of Hong Kong, HONG KONG

## Abstract

Ablation of glycosylated lysosomal membrane protein (GLMP, formerly known as NCU-G1) has been shown to cause chronic liver injury which progresses into liver fibrosis in mice. Both lysosomal dysfunction and chronic liver injury can cause metabolic dysregulation. *Glmp^gt/gt^* mice (formerly known as *Ncu-g1^gt/gt^*mice) were studied between 3 weeks and 9 months of age. Body weight gain and feed efficiency of *Glmp^gt/gt^* mice were comparable to wild type siblings, only at the age of 9 months the *Glmp^gt/gt^* siblings had significantly reduced body weight. Reduced size of epididymal fat pads was accompanied by hepatosplenomegaly in *Glmp^gt/gt^* mice. Blood analysis revealed reduced levels of blood glucose, circulating triacylglycerol and non-esterified fatty acids in *Glmp^gt/gt^* mice. Increased flux of glucose, increased *de novo* lipogenesis and lipid accumulation were detected in *Glmp^gt/gt^* primary hepatocytes, as well as elevated triacylglycerol levels in *Glmp^gt/gt^* liver homogenates, compared to hepatocytes and liver from wild type mice. Gene expression analysis showed an increased expression of genes involved in fatty acid uptake and lipogenesis in *Glmp^gt/gt^* liver compared to wild type. Our findings are in agreement with the metabolic alterations observed in other mouse models lacking lysosomal proteins, and with alterations characteristic for advanced chronic liver injury.

## Introduction

Glycosylated lysosomal membrane protein (GLMP, formerly known as NCU-G1) was first described as a nuclear protein, capable of regulating gene transcription [1]. Later studies identified GLMP as a *bona fide* lysosomal membrane protein [2–4]. The biological function of GLMP is unknown, but a high degree of conservation of the amino acid sequence indicates an important function [1]. Recently, we created and characterized a novel mouse model with no detectable expression of GLMP (*Glmp* ^*gt/gt*^ mouse, formerly known as *Ncu-g1* ^*gt/gt*^ mouse), and showed that the predominant phenotype is chronic liver injury which had developed into a well-established fibrosis by the age of 6 months [5]. Further analyses identified accumulation of iron in Kupffer cells [5], which has been shown to be associated with metabolic dysregulation [6]. Specific accumulation of iron in Kupffer cells has been shown to correlate with the severity of metabolic liver injury [7].

Lysosomal disorders are a group of congenital metabolic disorders caused by malfunctioning of a protein related to normal lysosomal function [8–13]. Most lysosomal disorders are characterized by impaired turnover of certain metabolites, which accumulate intralysosomally and affect normal lysosome and lysosome-related processes, such as autophagy [9–11]. Defective recycling of macromolecules through lysosomal pathways may cause insufficient recycling of metabolites and metabolic stress [11]. In fact, metabolic irregularities have been demonstrated in several mouse models with lysosomal disorders [14].

Liver fibrosis is a result of chronic liver injury, and is characterized by capillarization of sinusoids and redirection of blood directly from the portal tract to the hepatic vein [15–17]. The impaired perfusion of the liver and loss of fenestrations across the endothelial cell layer contribute to the imbalance in metabolite exchange between plasma and the liver [15]. The liver is also an important organ for regulation of glucose, lipid and protein metabolism, and chronic liver injury is known to cause alterations to glucose and lipid homeostasis [18].

Using the *Glmp* ^*gt/gt*^ mouse, a mouse model lacking this lysosomal membrane protein, and characterized by chronic liver injury and liver fibrosis, we assessed the metabolic consequences of GLMP ablation, focusing on liver.

## Materials and Methods

### Materials

[1-^14^C]oleic acid (58.2 mCi/mmol), D-[^14^C(U)]glucose (2.9 mCi/mmol) and [1-^14^C]acetic acid (56.0 mCi/mmol) were from PerkinElmer NEN (Boston, MA, US). Dulbecco's Modified Eagle Medium (DMEM), fetal bovine serum (FBS), Penicillin-Streptomycin (Pen-Strep), Fungizone and Dulbecco's phosphate-buffered saline (DPBS, with Mg^2+^ and Ca^2+^) were purchased from Life-Technologies (Carlsbad, CA, US). HEPES, L-carnitine, oleic acid (OA), glucose, bovine serum albumin (BSA), Triton X-100, sodium dodecyl sulfate (SDS) and protease inhibitor cocktail were from Sigma-Aldrich (St. Louis, MO, US). Sodium chloride and Tris Base were from VWR (Radnor, PA, US). Corning CellBIND tissue culture plates (12-well and 96-well plates) were obtained from Corning Life-Sciences (Schiphol-Rijk, The Netherlands). OptiPhase Supermix and UniFilter-96 GF/B were obtained from PerkinElmer (Shelton, CT, US). Thin layer chromatography plates were purchased from Merck (Darmstadt, Germany). Accu-Chek Aviva Nano Blood Glucose Meter System was purchased from Roche Applied Science (Mannheim, Germany). RNeasy Plus kit was obtained from Qiagen (Hilden, Germany). Brilliant III Ultra Fast SYBR Green qPCR Master Mix was obtained from Agilent Technologies (Santa Clara, CA, US) and LightCycler 480 SYBR Green I Master Mix was purchased from Roche Applied Science. Primers were designed and purchased from DNA Technology (Risskov, Denmark) and Life Technologies. Hypnorm was from VetaPharma (Leeds, UK). Midazolam was from Actavis (Parsippany, NJ, US). Animal diets were purchased from Scanbur (Karlslunde, Denmark). Protein assay reagent was purchased from Bio-Rad (Bio-Rad, Hercules, CA, US) and the content of proteins was determined using the Coomassie reagent [19]. All other chemicals used in this study were of standard commercial high-purity quality.

### Animal experiments

All animal experiments were reviewed and approved by the Norwegian Animal Research Authority, and performed according to national laws and regulations. Wild type (WT) and *Glmp* ^*gt/gt*^ mice [5] were housed in an approved animal facility with access to standard rodent chow and water *ad libitum* unless otherwise stated. Mice were 3 weeks old at the beginning of the feeding experiment. The body weight and food intake of a total of 27 male WT and 31 male *Glmp* ^*gt/gt*^ mice, housed in 12 different cages, and a total of 13 female WT and 20 female *Glmp* ^*gt/gt*^ mice, housed in 7 different cages were monitored at intervals of 7 days for 12 weeks, and then at 2, 3.5, 4.5, 6, 7.5 and 9 months of age. Feed efficiency was calculated as the ratio between body weight gain/week for each individual mouse and the average feed intake/mouse. Biological samples were collected at designated age points (1, 2, 3.5, 6, 7.5, and 9 months). Blood was obtained from randomly fed mice through cardiac puncture, and sera were collected after coagulation and centrifugation at 1500 *x g* for 20 minutes. Liver, spleen and epididymal fat pads (representing visceral adipose tissue) were dissected at selected time points, weighed and frozen in liquid nitrogen. The axillary and inguinal fat depots (representing subcutaneous adipose tissue) and interscapular brown adipose tissue were inspected at 1 and 4.5 months of age. All biological samples were stored at -80°C until further analysis.

### Resting blood glucose and serum and liver lipid levels

Resting blood glucose levels were measured in male WT and *Glmp* ^*gt/gt*^ mice (age = 5 months) fed *ad libitum* using Accu-Chek Aviva Nano Blood Glucose Meter System. Serum levels of non-esterified fatty acids (NEFA) were analyzed using NEFA-kit according to the supplier´s protocol (ILS Laboratories Scandinavia AS, Oslo, Norway). Triacylglycerol (TAG) was quantified in serum and whole liver homogenates using TG PAP 150-kit (BioMerieux, Marcy l’Etoile, France) according to the supplier’s protocol.

### Isolation of primary hepatocytes

Isolation of primary hepatocytes from WT and *Glmp* ^*gt/gt*^ mice (age = 4–5 weeks) was carried out by a two-step perfusion method as described [20]. Liver parenchymal and non-parenchymal cells were separated by differential centrifugation as described elsewhere [21]. Primary hepatocyte preparations with high viability (> 95%) were used in further studies.

### Uptake and oxidation of oleic acid and glucose

Determination of [^14^C]oleic acid (OA) or [^14^C]glucose uptake and oxidation has been described previously [22]. Primary hepatocytes were isolated as described above, and cultured in hepatocyte growth medium (DMEM high glucose, enriched with 20% FBS, Pen-Strep and Fungizone) on a 96-well microplate (80.000 cells/well) (Corning CellBIND). For measurements of OA or glucose uptake and oxidation, the growth medium was removed after 24 h and the cells were exposed to [^14^C]OA (0.5 μCi/mL, 100 μM) bound to BSA (40 μM) at a ratio of 2.5:1 in DPBS (with Mg^2+^ and Ca^2+^) with HEPES (10 mM), L-carnitine (1 mM) for 4 h or [^14^C]glucose (0.6 μCi/mL, 200 μM) in DPBS (with Mg^2+^ and Ca^2+^) with HEPES (10 mM), L-carnitine (1 mM) and BSA (7.2 μM) for 4 h. [^14^C]CO_2_ production and total uptake of OA or glucose (sum of CO_2_ and cell-associated radioactivity) were calculated using cell protein levels for standardization as described previously [22].

### Thin layer chromatography

Primary hepatocytes were isolated as described above, and cultured in hepatocyte growth medium in 12-well microplates (200.000 cells/well) (Corning CellBIND). The growth medium was removed after 24 h and the cells were exposed either to fatty acid medium, consisting of [^14^C]OA (0.5 μCi/mL, 100 μM) bound to BSA (40 μM) at a ratio of 2.5:1 in DPBS with HEPES (10 mM) and L-carnitine (1 mM) for 4 h, or to lipogenesis medium, consisting of [^14^C]acetate (0.5 μCi/mL, 100 μM) in DMEM enriched with glucose (5.5 mM) and BSA (10 μM) for 4 h. The media were collected and stored at -20°C until further analysis, and the remaining cells washed 3x with PBS before adding 250 μL H_2_O for cell lysis and solubilized cells were stored at -20°C. Cell-associated lipids were extracted with chloroform:methanol according to Folch et al. [23] and separated by thin layer chromatography as previously described [23]. The content of radiolabelled lipids was normalized against cell protein content.

### Analysis of gene expression

RNA extractions from mouse liver were carried out according to the manufacturer using RNeasy Plus kit. The expression of selected mRNA transcripts (S1 Table) was analyzed by qPCR using a LightCycler 480 (Roche Diagnostics, Manheim, Germany). PCR efficiencies were experimentally determined for each primer pair. Relative gene expression was calculated using the ΔΔCt-method, with Beta-actin and Eukaryotic translation elongation factor 2 as reference genes.

### Western blotting

Tritosomes from liver and kidneys from WT and *Glmp* ^*gt/gt*^ mice were isolated as described elsewhere [3]. Tritosome preparations (6 μg protein) were electrophoresed on NuPAGE 4–12% Bis-Tris Mini Gels (Life Technologies), and transferred onto PVDF membranes (Bio-Rad, Hercules, CA, USA). After blocking, membranes were incubated overnight at 4°C with rabbit anti-GLMP serum [3] or rabbit anti-LAMP1 (1:1000, C54H11, Cell Signaling, Beverly, MA, US). This was followed by 1 h incubation with goat anti-rabbit secondary antibody conjugated to horseradish peroxidase (1:4000, 65–6120, Life Technologies).

### Statistical methods

All results are expressed as mean ± SEM. Linear mixed models (LMM) were used to analyze overall differences in liver/body weight ratio, spleen/body weight ratio, serum TAG and serum NEFA levels, using IBM SPSS software (SPSS Inc. Chicago, IL, US). Other data were analyzed using two-tailed T-test (SigmaPlot, Systat Software Inc, Chicago, IL, US).

## Results

### Confirmation of GLMP ablation in *Glmp* ^*gt/gt*^ mice

In agreement with our previous report, analysis of lysosome-enriched fractions from mouse liver and kidneys after tyloxapol treatment [3] confirmed the lack of GLMP expression in *Glmp* ^*gt/gt*^ mice (S1 Fig).

### Similar body weight gain and feed intake in WT and *Glmp* ^*gt/gt*^ mice

**Fig 1:**
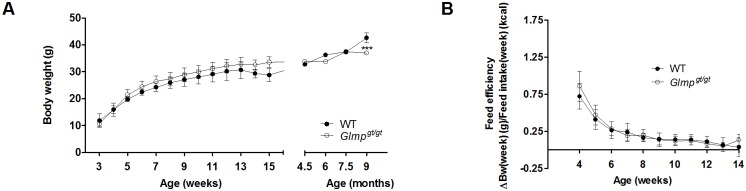
Loss of GLMP does not affect body weight gain and feed efficiency in young mice. (A) Body weight gain for male WT and *Glmp* ^*gt/gt*^ mice were monitored every week from 3 to 15 weeks of age, and then at 4.5, 6, 7.5 and 9 months of age. (B) The feed efficiencies of WT and *Glmp* ^*gt/gt*^ mice were monitored from 4 to 14 weeks of age (n = 27–31) (*** *p < 0*.*005* vs. WT). Values are presented as mean ± SEM.

Development of body weight in wild type (WT) and *Glmp* ^*gt/gt*^ mice was monitored from 3 weeks of age. As shown in Fig 1A, the body weight gains were similar for male WT and *Glmp* ^*gt/gt*^ mice up to the age of 7.5 months, but the *Glmp* ^*gt/gt*^ siblings were outgrown by WT mice at the age of 9 months. The feed efficiency was indistinguishable between the genotypes up to the age of 14 weeks (Fig 1B). Similar data were also obtained for female mice (data not shown).

### Smaller epididymal fat pads and overall hepatosplenomegaly in *Glmp* ^*gt/gt*^ mice

**Fig 2:**
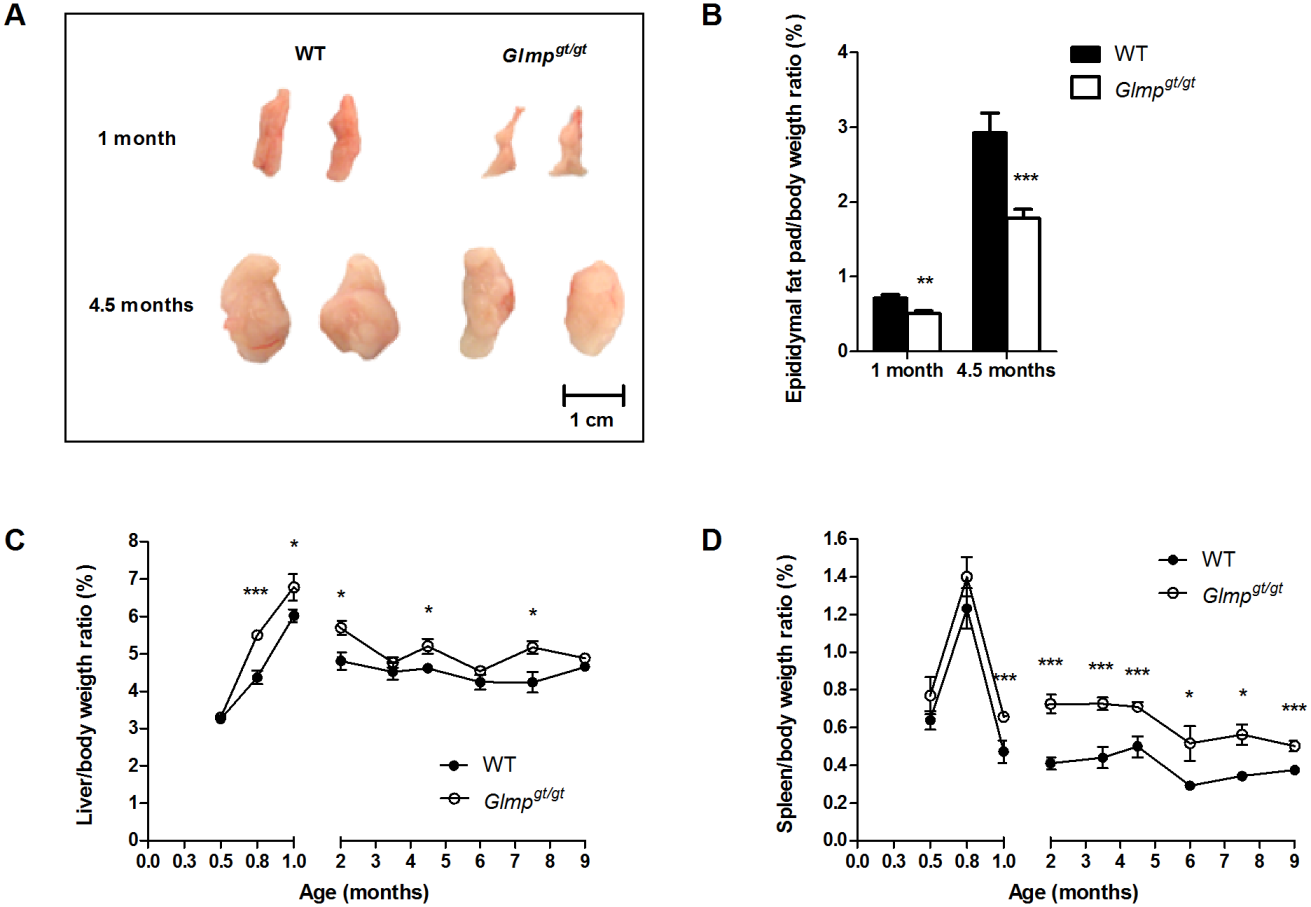
GLMP ablation causes reduced size of epididymal fat pads and hepatosplenomegaly. (A) Representative pictures of epididymal fat pads and (B) epididymal fat pads/body weight ratios in 1 (n = 4) and 4.5 months (n = 10–12) old WT and *Glmp* ^*gt/gt*^ mice. (C) Spleen/body weight ratios and (D) liver/body weight ratios in WT and *Glmp* ^*gt/gt*^ mice were examined at designated time-points between 2 weeks and 9 months of age (n = 10–12) (* *p < 0*.*05*, ** *p < 0*.*01*, *** p < 0.*005* vs. WT). Values are presented as mean ± SEM.

In spite of similar body weight, the *Glmp* ^*gt/gt*^ mice appeared leaner compared to the WT. Male WT and *Glmp* ^*gt/gt*^ mice were sacrificed at the age of one and 4.5 months, and their epididymal fat pads/body weight ratios were determined. As shown in Fig 2A and 2B, *Glmp* ^*gt/gt*^ mice had significantly smaller epididymal fat pads compared to WT at the age of one month, with an increased difference at 4.5 months of age. In contrast, the axillary and inguinal fat depots and the interscapular brown adipose tissue were comparable betweent he genotypes (data not shown). Next, we assessed the liver/body weight and spleen/body weight ratios in WT and *Glmp* ^*gt/gt*^ mice. The *Glmp* ^*gt/gt*^ liver was significantly enlarged at 0.8, 1, 2, 4.5 and 7.5 months of age compared to age-matched WT (Fig 2C), while *Glmp* ^*gt/gt*^ spleen was significantly enlarged at 1, 2, 3.5, 4.5, 6, 7.5 and 9 months of age compared to age-matched WT animals (Fig 2D).

### Decreased blood glucose, serum lipids and increased liver triacylglycerol (TAG) in *Glmp* ^*gt/gt*^ mice

**Fig 3:**
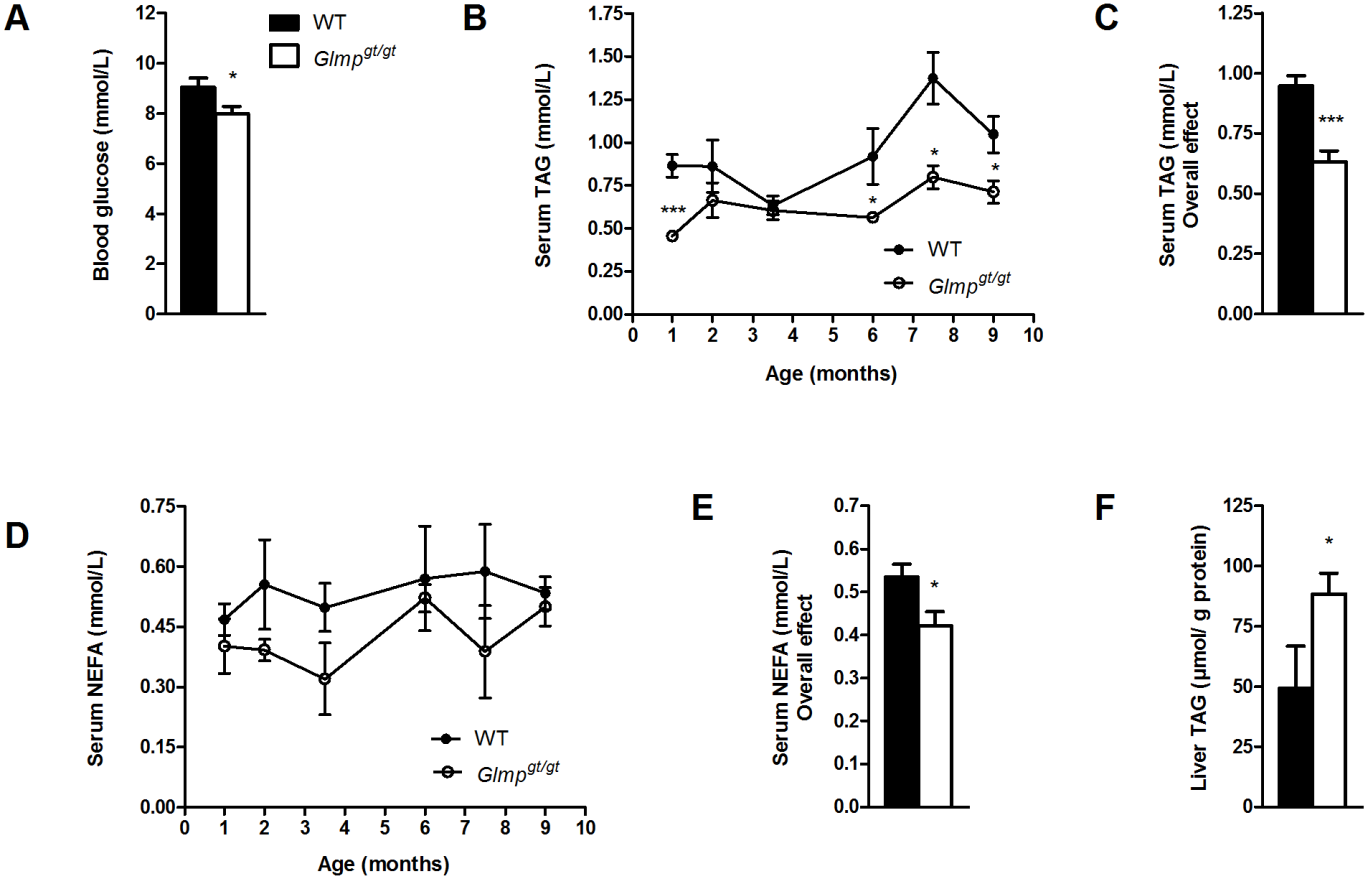
*Glmp* ^*gt/gt*^ mice have reduced blood glucose and circulating lipids, but increased liver triacylglycerol accumulation. (A) Blood glucose was analyzed in *ad libitum* fed 5 months old WT and *Glmp* ^*gt/gt*^ mice (n = 5). (B, C) The levels of circulating triacylglycerol (TAG) and (D, E) non-esterified fatty acids (NEFA) in WT and *Glmp* ^*gt/gt*^ mice were analyzed at designated time-points between 1 and 9 months of age (n = 4–8). (F) Liver TAG content was analyzed in 6 months old WT and *Glmp* ^*gt/gt*^ liver homogenates, and normalized to protein content (n = 6–7) (* *p < 0*.*05*, ** p < 0.*01*, *** *p < 0*.*005* vs. WT). Values are presented as mean ± SEM.

Five months old *Glmp* ^*gt/gt*^ mice had significantly reduced levels of blood glucose compared to WT mice when fed *ad libitum* (Fig 3A). Analysis of serum concentrations of triacylglycerol (TAG) in WT and *Glmp* ^*gt/gt*^ mice aged 1–9 months, showed an overall level that was significantly lower in the *Glmp* ^*gt/gt*^ mice (p < 0.001) (Fig 3B and 3C). A significant decrease was also found for serum non-esterified fatty acids (NEFA) concentrations in *Glmp* ^*gt/gt*^ compared to WT mice aged 1–9 months (p < 0.001) (Fig 3D and 3E). Liver content of TAG was measured in 6 months old WT and *Glmp* ^*gt/gt*^ mice. As shown in Fig 3F, there were significantly higher levels of liver TAG in *Glmp* ^*gt/gt*^ compared to WT mice.

### Changes in expression of metabolic genes in *Glmp* ^*gt/gt*^ liver

**Fig 4:**
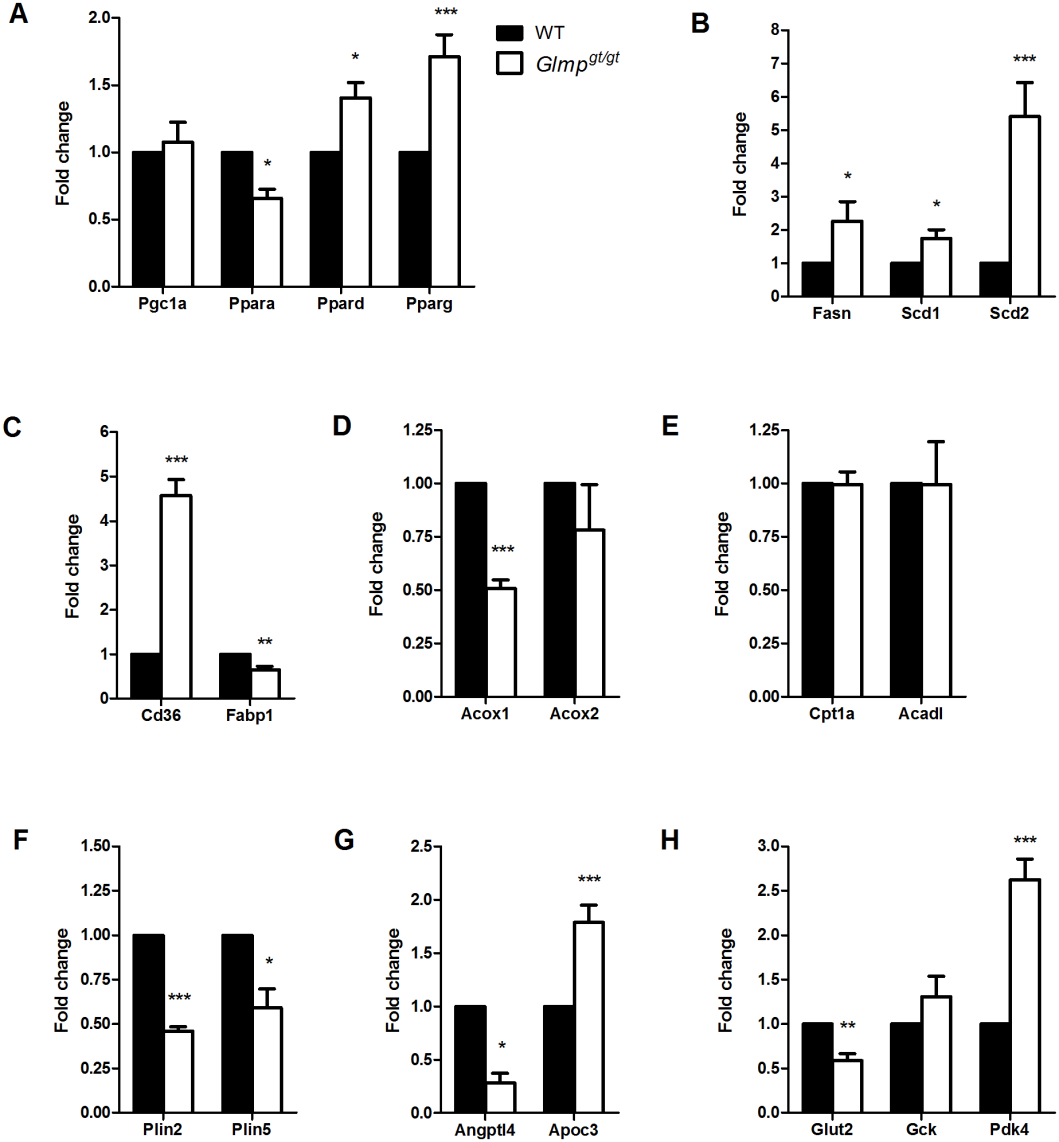
Altered expression of genes involved in metabolism in *Glmp* ^*gt/gt*^ liver. (A) qPCR analyses of 3.5 months old mouse livers from wild type (WT) and *Glmp* ^*gt/gt*^ mice showed altered expression of genes involved in metabolic regulation (*Pgc1a*, *Ppara*, *Ppard*, *Pparg*), (B) lipogenesis (*Fasn*, *Scd1*, *Scd2*), (C, D, E) lipid metabolism (*Cd36*, *Fabp1*, *Acox1*, *Acox2*, *Cpt1a*, *Acadl*) (F) lipid droplets (*Plin2*, *Plin5*), (G) very low-density lipoproteins (*Angptl4*, *Apoc3*) and (H) glucose metabolism (*Glut2*, *Gck*, *Pdk4*) in *Glmp* ^*gt/gt*^ liver (n = 4–8, * *p < 0*.*05*, ** p < 0.*01*, *** *p < 0*.*005* vs. WT). Values are presented as mean ± SEM.

Metabolic homeostasis is regulated by peroxisome proliferator-activated receptors (PPARs) [24]. In livers from 3.5 months old mice, GLMP ablation resulted in altered mRNA expression of the three PPAR isoforms. Fig 4A shows a decreased expression of *Ppara* and increased expression of *Ppard* and *Pparg* in *Glmp* ^*gt/gt*^ liver relative to WT animals. In contrast, the PPAR co-regulator *Pgc1a* was not differently expressed between the two genotypes. Genes involved in *de novo* lipogenesis (*Fasn*, *Scd1* and *Scd2*) were found to be significantly upregulated in *Glmp* ^*gt/gt*^ relative to WT liver (Fig 4B). The fatty acid transporter, *Cd36* showed a 4.6-fold increased expression in *Glmp* ^*gt/gt*^ liver, while cytoplasmic fatty acid binding protein *Fabp1* was significantly decreased compared to WT (Fig 4C). The expression of the initiator of peroxisomal fatty acid beta-oxidation, *Acox1* was significantly decreased in *Glmp* ^*gt/gt*^ liver, while no differences were found for the expression of *Acox2* (Fig 4D). Genes involved in mitochondrial fatty acid beta-oxidation (*Cpt1a* and *Acadl*) were not differently expressed between the two genotypes (Fig 4E). Furthermore, the expression of the lipid droplet associated proteins perilipin 2 and 5 (*Plin2* and *Plin5*) was significantly decreased in *Glmp* ^*gt/gt*^ liver (Fig 4F). A decreased expression of a lipoprotein lipase regulator, *Angptl4* was also detected in *Glmp* ^*gt/gt*^ liver, while the expression of *Apoc3*, a very low-density lipoprotein (VLDL) regulatory protein, was increased compared to WT (Fig 4G). With regard to genes involved in glucose metabolism, a decreased expression of the glucose transporter, *Glut2* was detected in *Glmp* ^*gt/gt*^ liver compared to WT. No differences in expression were detected for liver specific hexokinase (*Gck*), while the expression of *Pdk4*, an inhibitor of pyruvate dehydrogenase complex, was significantly upregulated in *Glmp* ^*gt/gt*^ liver compared to WT (Fig 4H).

### Altered glucose and fatty acid uptake and oxidation in *Glmp* ^*gt/gt*^ hepatocytes

**Fig 5:**
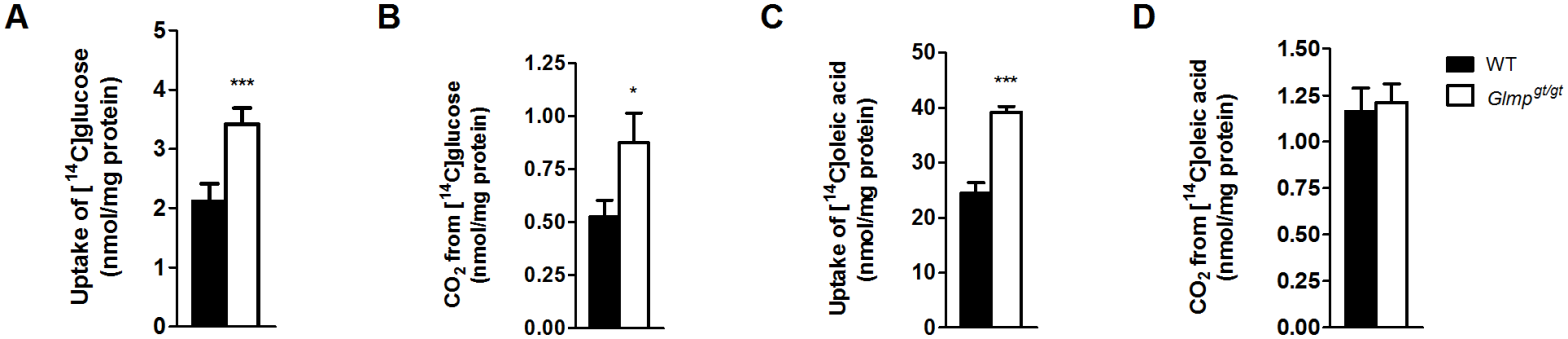
Glucose and oleic acid oxidation in WT and *Glmp* ^*gt/gt*^ hepatocytes. Overnight cultured primary hepatocytes from WT and *Glmp* ^*gt/gt*^ mice were incubated with [^14^C]glucose (0.6 μCi/mL, 200 μM) or [^14^C]oleic acid (OA) (0.5 μCi/mL, 100 μM) for 4 h. (A) Total [^14^C]glucose uptake, (B) [^14^C]glucose oxidation, (C) [^14^C]OA uptake and (D) [^14^C]OA oxidation were measured. Substrate uptake was calculated as the sum of cell-associated radioactivity and [^14^C]CO_2_ production. Substrate oxidation was measured as [^14^C]CO_2_ production. Results were normalized to protein content (n = 20 experiments, representing 4 individual mice/genotype, with 5 replicates each, * *p < 0*.*05*, *** *p < 0*.*005* vs. WT). Values are presented as mean ± SEM.

To assess whether GLMP ablation affects glucose and fatty acid metabolism in liver cells, hepatocytes were isolated from 4–6 weeks old *Glmp* ^*gt/gt*^ and WT mouse liver and exposed to either [^14^C]glucose or [^14^C]oleic acid (OA) for 4 h. Ablation of GLMP resulted in increased glucose uptake (Fig 5A) and oxidation (Fig 5B) in *Glmp* ^*gt/gt*^ hepatocytes. Exposure to [^14^C]OA revealed a significantly higher uptake of [^14^C]OA in *Glmp* ^*gt/gt*^ hepatocytes compared to WT cells (Fig 5C), while the oxidation capacity for OA was comparable between the two genotypes (Fig 5D).

**Fig 6:**
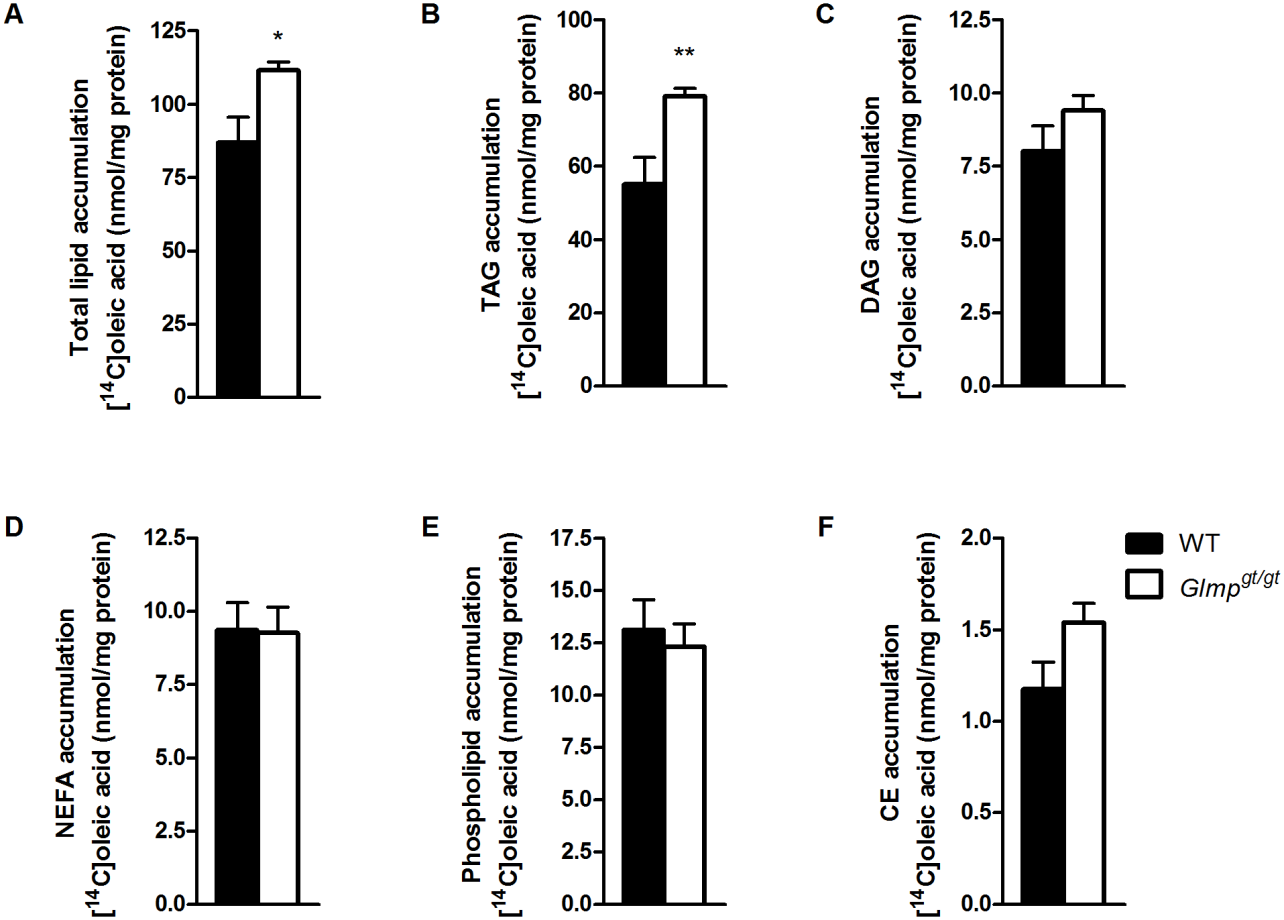
*Glmp* ^*gt/gt*^ hepatocytes incorporate more [^14^C]oleic acid into triacylglycerol. Overnight cultured primary hepatocytes from WT and *Glmp* ^*gt/gt*^ mice were incubated with [^14^C]oleic acid (0.5 μCi/mL, 100 μM) for 4 h. (A) The total amount of cell accumulated lipids, (B) triacylglycerol (TAG), (C) diacylglycerol (DAG), (D) non-esterified fatty acids (NEFA), (E) phospholipids and (E) cholesterol esters (CE) were analyzed using thin layer chromatography (n = 9 experiments, representing 3 individual mice/genotype, with 3 replicates each, ** *p < 0*.*01* vs. WT). Values are presented as mean ± SEM.

To further explore metabolism of OA, we performed thin layer chromatography on cell culture media and cell homogenates after 4 h exposure to [^14^C]OA. No significant difference in secretion of triacylglycerol (TAG) into the media could be detected (data not shown), but there was a significant increase of accumulated total lipids in *Glmp* ^*gt/gt*^ hepatocytes compared to WT hepatocytes (Fig 6A). This difference was primarily contributed to by a significant increase in TAG accumulation (Fig 6B). Incorporation of labelled OA in other lipids assayed, diacylglycerol (DAG) (Fig 6C), non-esterified fatty acids (NEFA) (Fig 6D), phospholipids (Fig 6E), and cholesterol esters (CE) (Fig 6F) did not significantly differ between *Glmp* ^*gt/gt*^ and WT hepatocytes.

### Increased de novo lipogenesis in *Glmp* ^*gt/gt*^ hepatocytes

**Fig 7:**
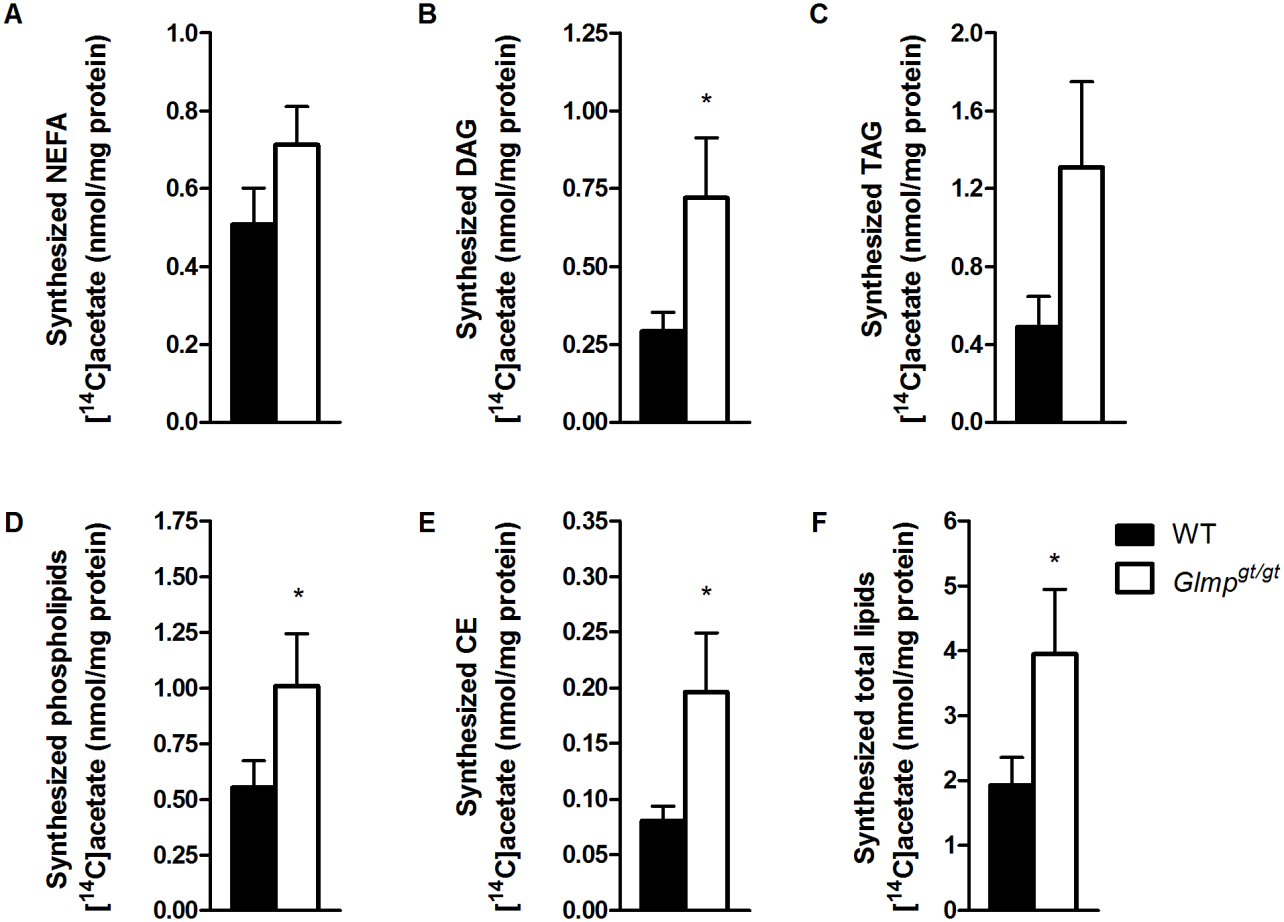
Increased *de novo* lipogenesis in *Glmp* ^*gt/gt*^ hepatocytes. Overnight cultured primary hepatocytes from WT and *Glmp* ^*gt/gt*^ mice were incubated with [^14^C]acetate (0.5 μCi/mL, 100 μM) for 4 h. (A) The amount of synthesized non-esterified fatty acids (NEFA), (B) diacylglycerol (DAG), (C) triacylglycerol (TAG), (D) phospholipids, (E) cholesterol esters (CE) and (F) total lipids were analyzed using thin layer chromatography (n = 8 experiments, representing 4 individual mice/genotype, with 2 replicates each, * *p < 0*.*05* vs. WT). Values are presented as mean ± SEM.

Primary hepatocytes from WT and *Glmp* ^*gt/gt*^ mice were exposed to [^14^C]acetate for 4 h, and thin layer chromatography was performed on cell homogenates in order to assess *de novo* lipogenesis. The levels of newly synthesized NEFA showed no significant difference between the two genotypes (Fig 7A), but the incorporation into DAG was found to be significantly elevated (Fig 7B). A clear tendency for increased synthesis of TAG was also observed for *Glmp* ^*gt/gt*^ hepatocytes (Fig 7C), which, however, did not reach statistical significance. Higher incorporation of labelled acetate into PL (Fig 7D) and CE (Fig 7E) was also detected in *Glmp* ^*gt/gt*^ hepatocytes. Taken together, these results indicate increased lipogenesis in *Glmp* ^*gt/gt*^ hepatocytes compared to WT cells, which was also reflected in the total amount of synthesized lipids (Fig 7F).

## Discussion

In this study, we showed that wild type (WT) and *Glmp* ^*gt/gt*^ mice initially had similar growth rates. The feed efficiency was also comparable for the two genotypes up to the age of 14 weeks. Yet the epididymal fat pads were significantly smaller in *Glmp* ^*gt/gt*^ siblings compared to WT mice at both one and 4.5 months of age. Other fat depots such as the axillary and inguinal white adipose tissue and the interscapular brown adipose tissue, however, were comparable between the genotypes. Hepatosplenomegaly contributed to the similar body weights until the age of 9 months when the *Glmp* ^*gt/gt*^ siblings had significantly reduced body weight. The reduced sizes of the epididymal fat pads were reflected in lower levels of circulating triacylglycerol (TAG) and non-esterified fatty acids (NEFA) in *Glmp* ^*gt/gt*^ mice. In addition, the *Glmp* ^*gt/gt*^ mice had lower blood glucose concentration. Gene expression analysis using liver mRNA from WT and *Glmp* ^*gt/gt*^ mice indicated changes in both glucose and lipid metabolic pathways, and biochemical analysis of whole liver homogenates detected higher TAG levels in *Glmp* ^*gt/gt*^ liver. Exposing isolated primary hepatocytes from WT and *Glmp* ^*gt/gt*^ mice to radiolabelled oleic acid (OA) showed that the *Glmp* ^*gt/gt*^ hepatocytes accumulated significantly more OA than WT hepatocytes, and that the increased OA taken up was stored as TAG. Exposure to radiolabelled glucose and acetate revealed a higher oxidation of glucose and increased *de novo* lipogenesis, respectively, in *Glmp* ^*gt/gt*^ hepatocytes compared to WT cells.

In most lysosomal disorders, the primary lysosomal dysfunction induces secondary alterations in other metabolic pathways [25], some of which may be adaptive changes in order to provide metabolites for *de novo* synthesis of macromolecules, because lysosomal dysfunction might inhibit adequate recycling of metabolites [14, 26]. As a consequence, reduced adiposity and body weight, possibly caused by higher tissue demand for metabolites, has been observed in several mouse models with lysosomal disorders [14, 26]. However, the body weight of younger *Glmp* ^*gt/gt*^ mice was comparable to that of their WT siblings since the reduced adiposity was compensated by hepatosplenomegaly. Similar observations have been reported for the mouse models for mucopolysaccharidosis (MPS) type I and IIIB [14]. Interestingly, as seen in the *Glmp* ^*gt/gt*^ mouse, reduced adiposity, reduced blood glucose, reduced lipid levels and hepatomegaly were also reported in these mice [14]. A similar feed efficiency between WT and *Glmp* ^*gt/gt*^ mice suggested that the *Glmp* ^*gt/gt*^ mice did not suffer from malabsorption as was also shown for mouse models with other lysosome deficiencies [14].

We have recently established that the *Glmp* ^*gt/gt*^ mice suffer from a mild chronic liver injury, as indicated by the modest increase in serum transaminase levels. However, by the age of 6 months, the sustained liver injury led to a well-established fibrosis with increased inflammation and oxidative stress [5], conditions known to disturb the liver's capacity for regulating glucose, lipid and protein homeostasis [18]. As the liver is an important organ for glucose homeostasis [27], the small decrease in resting blood glucose may be caused by increased glucose flow to the liver. Somewhat conflicting studies have been reported regarding glucose uptake capacity in fibrotic liver, which has been ascribed to differences in experimental setup [28–32]. A recent study using primary hepatocytes isolated from early, and advanced fibrotic rat livers, demonstrated a gradual decrease in mitochondrial function [33], an established secondary effect of advanced liver fibrosis [34]. Hepatocytes from early fibrotic livers increased the glycolytic flux in order to compensate for impaired ATP-production by oxidative phosphorylation [33]. Although the ATP-production capacity of *Glmp* ^*gt/gt*^ mitochondria needs to be further elucidated, we showed by using radiolabelled glucose that the *Glmp* ^*gt/gt*^ hepatocytes consumed significantly higher levels of glucose compared to WT hepatocytes. Increased flow of glucose to the liver is known to stimulate the expression of lipogenic genes [35]. By using radiolabelled acetate, we showed that *de novo* lipogenesis was induced in *Glmp* ^*gt/gt*^ hepatocytes, an expected result given the increased expression of lipogenic genes in *Glmp* ^*gt/gt*^ liver.

Unexpectedly, the expression of *Pdk4* was significantly increased in *Glmp* ^*gt/gt*^ liver, suggesting an inhibition of the pyruvate dehydrogenase complex, an event usually associated with inhibition of *de novo* lipogenesis and activation of gluconeogenesis [36]. An increase in *Pdk4* expression has been reported in two independent mouse models of liver fibrosis, resulting from chemical-induced liver damage [37] and chronic biliary injury [38]. Interestingly, the increased *Pdk4* expression in the chemical-induced model was not accompanied by increased expression of gluconeogenic genes [37]. Similarily, the *Mdr2* ^-/-^ mouse liver (model for chronic biliary injury [39]) had increased expression of lipogenic genes, and developed steatotic nodules [38]. In both studies, the increased *Pdk4* expression was explained as preventive adaptations to the hepatic injury [37, 38]. Increased *Pdk4* expression in the *Glmp* ^*gt/gt*^ liver is likely to be an adaptive response to the chronic liver injury, although increased glucose flux through the pentose phosphate pathway, which contributes to the antioxidant defense, and NADPH production required for *de novo* lipogenesis [40] is also a possibility.

Abnormal accumulation of lipids in the liver is a known consequence of dysregulation of metabolism [41]. However, in our initial report, histological analysis failed to reveal obvious steatosis in *Glmp* ^*gt/gt*^ liver [5]. Here, using a biochemical approach, we showed that *Glmp* ^*gt/gt*^ mice accumulated slightly more liver TAG than WT animals. The small, relative difference might be undetectable using histological methods. In support of the elevated TAG accumulation in *Glmp* ^*gt/gt*^ liver, the increased expression of PPARγ as seen in the *Glmp* ^*gt/gt*^ liver is a hallmark of hepatic steatosis [42], as it stimulates expression of lipogenic genes [18, 43] and the fatty acid transporter, CD36 [44]. Increased *Cd36* mRNA expression has been found to correlate with increased hepatic TAG content in different models of hepatic steatosis [45–47]. After exposure to radiolabelled OA, an increased fatty acid uptake concomitant with increased storage as TAG in *Glmp* ^*gt/gt*^ hepatocytes was observed, supporting the observed TAG accumulation in *Glmp* ^*gt/gt*^ liver. Apart from increased fatty acid uptake by hepatocytes and *de novo* lipogenesis, compromised assembly and secretion of VLDL particles can contribute to lipid accumulation [18, 41, 48, 49]. In our experimental setup, no significant decrease of TAG secretion was detected for *Glmp* ^*gt/gt*^ hepatocytes. The increased expression of *Apoc3*, as detected in *Glmp* ^*gt/gt*^ liver is probably a result of decreased PPARα expression and increased influx of glucose [50]. *Apoc3* expression is usually positively correlated with plasma TAG levels [51, 52]. However, ApoC3-containing lipoprotein secretion is stimulated by elevated levels of plasma NEFA [53], which were decreased in *Glmp* ^*gt/gt*^ mice.

The detected liver steatosis in *Glmp* ^*gt/gt*^ mice may have been promoted by chronic inflammation [54, 55]. In our previous study, increased expression of inflammatory cytokines, including tumor necrosis factor α (TNFα) and interleukin 1β (IL-1β) were detected in *Glmp* ^*gt/gt*^ liver [5]. Kupffer cells, activated by liver injury, have been shown to be essential for the development of diet-induced hepatic steatosis in rats, as they alter metabolic pathways in hepatocytes through TNFα secretion [56]. TNFα promotes the lipogenic inducer sterol regulatory element binding protein-1c (SREBP1c) [57] and the protein expression of CD36 [58]. In addition, TNFα stimulates lipolysis in peripheral adipose tissue to release free fatty acids into the circulation [59]. The reduced adipose tissue size and reduced levels of circulating fatty acids observed in *Glmp* ^*gt/gt*^ mice may be due to prolonged stimulation of adipose tissue lipolysis. Finally, activated Kupffer cells secrete IL-1β, which downregulates the expression of PPARα [60], a positive regulator of lipid catabolism [61].

The recent identification of a lysosomal nutrient sensing machinery (LYNUS) has provided a direct link between intralysosomal nutrient levels and the expression of metabolic genes [62, 63]. In periods of nutrient scarcity, LYNUS releases the transcription factor EB into the nucleus where it promotes the expression of genes involved in autophagy and lysosomal biogenesis, in addition to genes involved in fatty acid *β*-oxidation through induced expression of PPARα and PGC1α [63–65]. A significant decrease in *Ppara* expression was detected in *Glmp* ^*gt/gt*^ liver, and we might hypothesize that ablation of the lysosomal membrane protein, GLMP, influences normal LYNUS function, a secondary effect observed in many lysosomal disorders [11], thus affecting normal *Ppara* expression and lipid turnover. Dysregulation of metabolism in *Glmp* ^*gt/gt*^ liver might in turn affect adipose tissue. However, one of the limitations of the present study is the inability to dismiss the metabolic alterations in *Glmp* ^*gt/gt*^ liver as secondary effects. We are currently establishing a cell-type-specific transgenic mouse model in order to address this question in future studies.

In summary, we showed that *Glmp* ^*gt/gt*^ mice have a similar body weight gain and feed efficiency as WT mice until 9 months of age. Similar to some other mouse models with a dysfunctional lysosomal protein, a reduced adiposity and an liver fibrosis were also detected in *Glmp* ^*gt/gt*^ mice [5]. Analysis of serum glucose and lipid levels showed a significant decrease in *Glmp* ^*gt/gt*^ mice. In addition to increased hepatic TAG content and changes in the expression of metabolic genes in *Glmp* ^*gt/gt*^ liver, our data strongly indicate metabolic dysregulation in *Glmp* ^*gt/gt*^ mice. Analysis using primary hepatocytes supported the findings in *Glmp* ^*gt/gt*^ liver, and showed enhanced uptake of fatty acids, increased *de novo* lipogenesis, and higher consumption of glucose in *Glmp* ^*gt/gt*^ hepatocytes. Ablation of the lysosomal membrane protein GLMP has recently been shown to cause chronic liver injury in mice [5]. In this study, we demonstrated that metabolic dysregulation is a likely contributor to the pathophysiology following GLMP ablation.

## Supporting Information

S1 Fig Verification of disrupted GLMP expression in *Glmp* ^*gt/gt*^ mice. Lysosome-enriched fractions from mouse kidney and liver after tyloxapol treatment were used to verify the ablation of GLMP expression in *Glmp* ^*gt/gt*^ mice. LAMP1 served as loading control.
(TIF)

S1 Table Primers used in qPCR. (DOC)
