# Supplementary figures and images for: Lack of the Lysosomal Membrane Protein, GLMP, in Mice Results in Metabolic Dysregulation in Liver
Source: PLoS One. 2015 Jun 5;10(6):e0129402. doi: 10.1371/journal.pone.0129402 (PMC4457871; doi:10.1371/journal.pone.0129402)

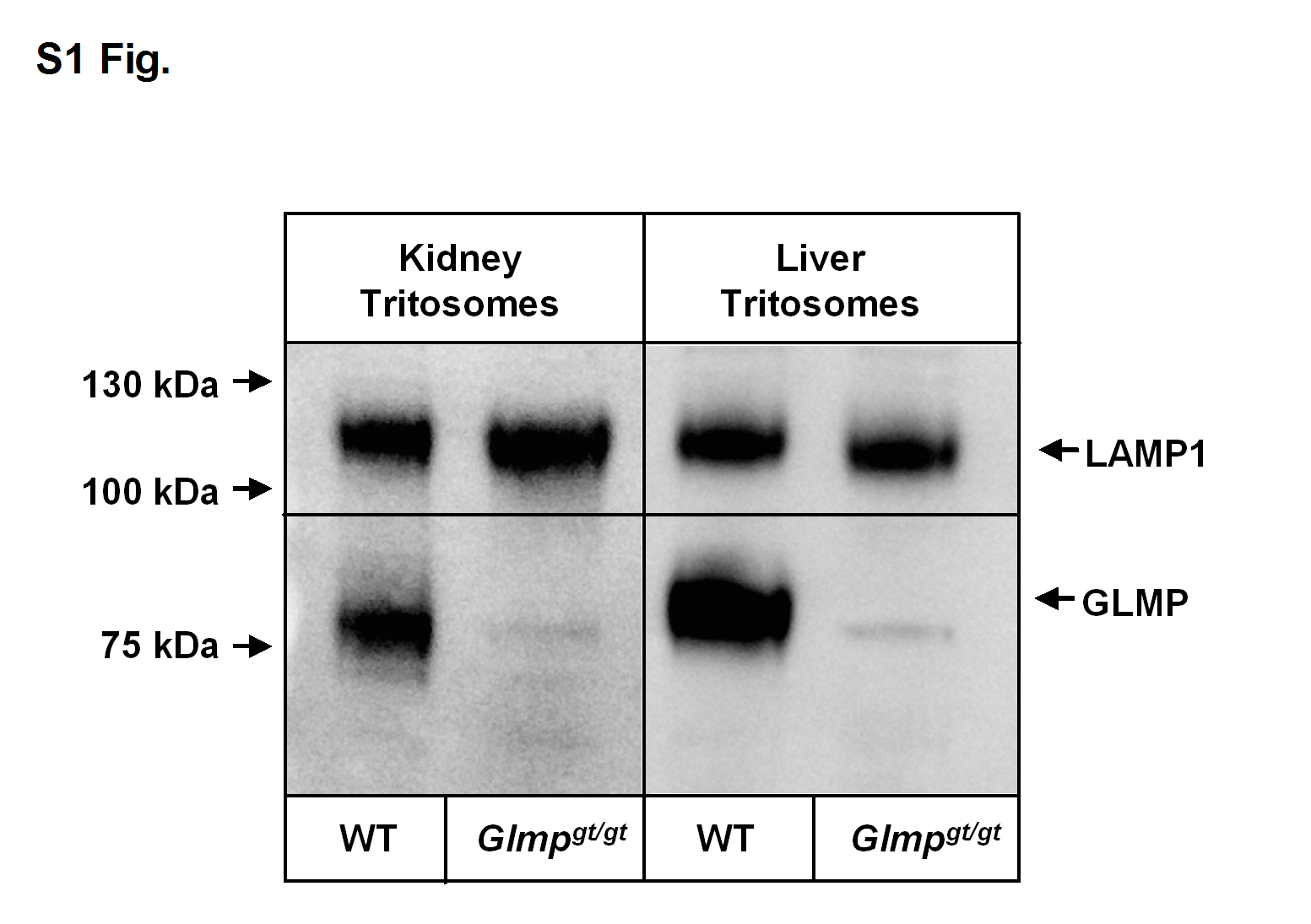

Supplement: S1 Fig — Lysosome-enriched fractions from mouse kidney and liver after tyloxapol treatment were used to verify the ablation of GLMP expression in Glmp gt/gt mice. LAMP1 served as loading control. (TIF) [file pone.0129402.s001.tif]
