# Supplementary material for: Lack of the Lysosomal Membrane Protein, GLMP, in Mice Results in Metabolic Dysregulation in Liver
Source: PLoS One. 2015 Jun 5;10(6):e0129402. doi: 10.1371/journal.pone.0129402 (PMC4457871; doi:10.1371/journal.pone.0129402)
Supplement: S1 Table — (DOC) [file pone.0129402.s002.doc]

**S1 Table.** **Primers used in qPCR.**

| Target gene | Description | 5'-Primer | 3'-Primer |
| --- | --- | --- | --- |
| *Acadl* | Acyl-CoA Dehydrogenase (Long Chain) | GGGAAGAGCAAGCGTACTCC | TCTGTCATGGCTATGGCACC |
| *Acox1* | Acyl-CoA Oxidase 1 | CATGGAACTCATCTTCGAGGCTTGG | AAAGGCATGTAACCCGTAGCACTCC |
| *Acox2* | Acyl-CoA Oxidase 2 | TCAGTCCGTCATGTATCTCGCCACA | CTGTGTTGCATCTCTTATGAGCCTG |
| *Angptl4* | Angiopoietin-like 4 | CTGGGTGCCACCAATGTTTC | GTCCCCACGGAGGTCATG |
| *Apoc3* | Apolipoprotein C-III | AGCGTGCAGGAGTCCGATATA | TTAGAATCCCAGAAGCCGGTG |
| *Cd36* | CD36 Molecule (Thrombospondin Receptor) | GGCCAAGCTATTGCGACAT | CAGATCCGAACACAGCGTAGA |
| *Cpt1a* | Carnitine palmitoyltransferase 1A | CCTGCTAGTACCCAAGTGTGAC | AAGCCCACTCTTCTGCCTT |
| *Fabp1* | Fatty acid binding protein 1 (Liver) | AAACTCACCATCACCTATGGAC | ATTGAGTTCAGTCACGGACTTT |
| *Fasn* | Fatty acid synthase | TCCTGGAACGAGAACACGATCT | AGAGACGTGTCACTCCTGGACTT |
| *Gck* | Glucokinase | TCAGCCGGATGCAGAAGGA | GCAACATCTTTACACTGGCCT |
| *Glut2* | Glucose Transporter Type 2 (Liver) | GGCTAATTTCAGGACTGGTT | TTTCTTTGCCCTGACTTCCT |
| *Pdk4* | Pyruvate Dehydrogenase Kinase 4 | AAAGGACAGGATGGAAGGAATCA | TTTTCCTCTGGGTTTGCACAT |
| *Pgc1a* | Peroxisome proliferator-activated receptor gamma, coactivator 1 alpha | ATTGTTCGATGTGTCGCCTTC | TGAACGAGAGCGCATCCTTT |
| *Plin2* | Perilipin 2 | CAGCCAACGTCCGAGATTG | CATGGTAGTCGTCACCACATCCT |
| *Plin5* | Perilipin 5 | TCCGCCATCTCGCCTATG | CAGCTGGGCCAGCATCTC |
| *Ppara* | Peroxisome proliferator-activated receptor alpha | TCTTCACGATGCTGTCCTCCT | GGAACTCGCCTGTGATAAAGC |
| *Ppard* | Peroxisome proliferator-activated receptor delta | TCCAGAAGAAGAACCGCAACA | GGATAGCGTTGTGCGACATG |
| *Pparg* | Peroxisome proliferator-activated receptor gamma | CAGGCCGAGAAGGAGAAGCT | GGCTCGCAGATCAGCAGACT |
| *Scd1* | Stearoyl-CoA desaturase 1 | ATGTGGCTTTGGCTGATCCAT | ACTTGCCCATGTCTCTGGTGT |
| *Scd2* | Stearoyl-CoA desaturase 2 | ACTGTGACTCAAGTTCAACTCTTGAAA | TGCCCACAAATTGAGGATAGC |
| *eEF2* | Eukaryotic translation elongation  factor 2 | CCATCGCTGAACGCATCAAG | CAGGCCAGAACCAAAGCCTA |
| *β-actin* | Beta-actin | AGCCATGTACGTAGCCATCC | GCTGTGGTGGTGAAGCTGTA |
|  |  |  |  |
